# Urinary Levels of SARS-CoV-2 Nucleocapsid Protein Associate With Risk of AKI and COVID-19 Severity: A Single-Center Observational Study

**DOI:** 10.3389/fmed.2021.644715

**Published:** 2021-05-25

**Authors:** Désirée Tampe, Samy Hakroush, Mark-Sebastian Bösherz, Jonas Franz, Heike Hofmann-Winkler, Stefan Pöhlmann, Stefan Kluge, Onnen Moerer, Christine Stadelmann, Philipp Ströbel, Martin Sebastian Winkler, Björn Tampe

**Affiliations:** ^1^Department of Nephrology and Rheumatology, University Medical Center Göttingen, Göttingen, Germany; ^2^Institute of Pathology, University Medical Center Göttingen, Göttingen, Germany; ^3^Institute of Neuropathology, University Medical Center Göttingen, Göttingen, Germany; ^4^Max Planck Institute for Experimental Medicine, Göttingen, Germany; ^5^Campus Institute for Dynamics of Biological Networks, University of Göttingen, Göttingen, Germany; ^6^Infection Biology Unit, German Primate Center, Leibniz Institute for Primate Research Göttingen, Göttingen, Germany; ^7^Faculty of Biology and Psychology, University Göttingen, Göttingen, Germany; ^8^Department of Intensive Care Medicine, University Medical Center Hamburg-Eppendorf, Hamburg, Germany; ^9^Department of Anesthesiology, Emergency and Intensive Care Medicine, University Medical Center Göttingen, Göttingen, Germany

**Keywords:** severe acute respiratory syndrome coronavirus-2, SARS-CoV-2 disease 2019, acute kidney injury, intensive care, risk prediction

## Abstract

**Background:** Acute kidney injury (AKI) is very common in severe acute respiratory syndrome coronavirus-2 (SARS-CoV-2) disease 2019 (COVID-19) and considered as a risk factor for COVID-19 severity. SARS-CoV-2 renal tropism has been observed in COVID-19 patients, suggesting that direct viral injury of the kidneys may contribute to AKI. We examined 20 adult cases with confirmed SARS-CoV-2 infection requiring ICU supportive care in a single-center prospective observational study and investigated whether urinary markers for viral infection (SARS-CoV-2 N) and shedded cellular membrane proteins (ACE2, TMPRSS2) allow identification of patients at risk for AKI and outcome of COVID-19.

**Objectives:** The objective of the study was to evaluate whether urinary markers for viral infection (SARS-CoV-2 N) and shedded cellular membrane proteins (ACE2, TMPRSS2) allow identification of patients at risk for AKI and outcome of COVID-19.

**Results:** Urinary SARS-CoV-2 N measured at ICU admission identified patients at risk for AKI in COVID-19 (HR 5.9, 95% CI 1.4–26, *p* = *0.0095*). In addition, the combination of urinary SARS-CoV-2 N and plasma albumin measurements further improved the association with AKI (HR 11.4, 95% CI 2.7–48, *p* = *0.0016*). Finally, combining urinary SARS-CoV-2 N and plasma albumin measurements associated with the length of ICU supportive care (HR 3.3, 95% CI 1.1–9.9, *p* = *0.0273*) and premature death (HR 7.6, 95% CI 1.3–44, *p* = *0.0240*). In contrast, urinary ACE2 and TMPRSS2 did not correlate with AKI in COVID-19.

**Conclusions:** In conclusion, urinary SARS-CoV-2 N levels associate with risk for AKI and correlate with COVID-19 severity.

## Introduction

Acute kidney injury (AKI) is very common in severe acute respiratory syndrome coronavirus-2 (SARS-CoV-2) disease 2019 (COVID-19), particularly among patients requiring intensive care unit (ICU) supportive care and is considered as an independent risk factor for premature death (1–5). Rates of reported AKI vary considerably with higher rates reported in countries outside of China (6). Therefore, there is an urgent need for early identification of patients at risk for AKI in COVID-19. SARS-CoV-2 renal tropism and detection of SARS nucleocapsid protein (SARS-CoV-2 N) in renal tubules has been described in COVID-19 patients, suggesting that direct viral injury of the kidneys may contribute to AKI (7–9). This is in line with two autopsy studies suggesting renal SARS-CoV-2 infection as a possible cause of AKI in COVID-19 (7, 8). SARS-CoV-2 renal tropism has been in part attributed to the intrarenal presence of cellular membrane proteins essential for viral entry including angiotensin converting enzyme 2 (ACE2) and transmembrane protease serine subtype 2 (TMPRSS2) (9–11). Based on the assumption that renal injury is caused by direct viral infection of the kidneys, it remains unclear if markers for viral infection (SARS-CoV-2 N) and shedded cellular membrane proteins (ACE2, TMPRSS2) in urinary samples are useful for early identification of COVID-19 patients at risk for AKI. Therefore, we investigated whether risk for AKI in COVID-19 is associated with urinary SARS-CoV-2 N, ACE2, and TMPRSS2 levels, thus allowing for simple and fast identification of patients at risk. We report that urinary SARS-CoV-2 N levels are linked to AKI and COVID-19 severity.

## Materials and Methods

### Study Populations

All patients with PCR-based SARS-CoV-2 infection confirmed by nasopharyngeal swabs were admitted to the Department of Anesthesiology, Emergency and Intensive Care Medicine, University Medical Center Göttingen, Germany and those requiring ICU supportive care were included. The institutional ethical board of the University Medical Center Göttingen, Germany approved the study (reference number 25/4/19Ü), and informed written consent was obtained. All urinary samples were collected at the University Medical Center Göttingen, Germany between March and June 2020. Urinary samples from severe septic shock patients collected in 2015 served as control and have been previously described and recruited at the Department of Intensive Care Medicine, University Medical Center Hamburg-Eppendorf, Germany (12). The study was approved by the ethical committee Hamburg (Aerztekammer Hamburg, reference number PV4550). A detailed Strengthening the Reporting of Observational Studies in Epidemiology (STROBE) flow chart of patient disposition is shown in Supplementary Figure 1.

### Definitions

Comorbidities were assessed using reported information by hospitals or private practices. Arterial hypertension, diabetes, hyperlipoproteinemia, or obesity was considered as metabolic diseases. Liver diseases, malignancies, and autoimmune diseases were considered as other diseases. Group allocation into AKI was defined according to KDIGO by laboratory findings, oliguria, or the need for dialysis. The simplified acute physiology score (SAPS) II and sequential organ failure assessment (SOFA) score were calculated according to published guidelines (13, 14). Requirement of intensive care treatment was defined at admission and calculated by the time between admission to the intensive care unit (ICU) or intermediate care unit (IMC) and relocation to the non-ICU/non-IMC medical ward; all patients required critical care treatment >24 h.

### Urinary ELISA Measurements

Investigators were blinded to clinical data collection and urinary enzyme-linked immunosorbent assay (ELISA) measurements. According to the manufacturer's protocols, the urinary levels of SARS-CoV-2 N (KIT40588, Sino Biological), human ACE2 (NBP2-78734, Novus Biologicals), and human TMPRSS2 (NBP2-89170, Novus Biologicals) were analyzed. In brief, 100 μL of native patient urine was analyzed by sandwich ELISA using precoated plates with antibodies specific to SARS-CoV-2 N, human ACE2, and human TMPRSS2. After adding a biotinylated detection antibody, avidin-horseradish peroxidase conjugate was added successively to each microplate well and incubated. After washing, the substrate solution was added to each well. The enzyme–substrate reaction was terminated by addition of a stop solution, and the optical density was measured spectrophotometrically at a wavelength of 450 nm. Measurements were done in triplicates for each urinary sample and compared to the standard curve. Negative test results were declared as not detectable. Photomicrographs of urinary SARS-CoV-2 N ELISA measurements in 20 cases with confirmed SARS-CoV-2 infection requiring ICU supportive care are shown in Supplementary Figure 2.

### SARS-CoV-2 RNA Detection

Tissues were dissolved in TRIzol (Zymo) and shredded using QiaShredder (Qiagen). RNA was extracted according to the manufacturer's protocol. RNA (0.5–1 μg) was added to the final PCR reaction. SARS-CoV-2 genome equivalents were detected by quantitative RT–PCR targeting the SARS-CoV-2 E gene as previously reported using the following primers: E_Sarbeco_F, ACAGGTACGTTAATAGTTAATAGCGT; E_Sarbeco_P1, FAM-ACACTAGCCATCCTTACTGCGCTTCG-BBQ; E_Sarbeco_R, ATATTGCAGCAGTACGCACACA (15, 16). The quantitative RT-PCR experiment and data processing were carried out using the LightCycler 480 Real-Time PCR System (Roche) and LightCycler 480 Software (version 1.5, Roche Molecular Systems). Absolute quantification was performed using SARS-CoV-2-specific *in vitro*–transcribed RNA standards, as previously described (15, 16).

### Sample Size and Statistical Analysis

At the time of the study design, data on markers for viral infection (SARS-CoV-2 N) and shedded cellular membrane proteins (ACE2, TMPRSS2) in urinary samples of AKI in COVID-19 patients were lacking. Hence, a convenience sample of 20 consecutive patients was chosen to provide a timely report. Variables were tested for normal distribution using the Shapiro–Wilk test. Non-normally distributed continuous variables are expressed as median and interquartile range (IQR); categorical variables are presented as frequency and percentage. Statistical comparisons were not formally powered or prespecified. For group comparisons, the Mann–Whitney *U*-test was used to determine differences in medians. Non-parametric between-group comparisons were performed with Pearson's chi-square test. Correlation between parameters was calculated by Spearman's rank correlation and is shown by heatmap reflecting the mean values of Spearman's ρ; asterisks indicate *p* < *0.05*. We retained those covariates found to be significantly associated with AKI in a multivariable regression model (limiting the model to four covariates to avoid model overfit). To establish a cutoff for each parameter, the ability of prognostic factors to discriminate groups was evaluated by receiver operator curves (ROC) and the area under the curve (AUC), as well as sensitivity and specificity. Sensitivity and specificity were based on selection of the cutoff point on the ROC that maximized Youden's index (sensitivity+specificity-1); comparison of survival curves was performed with log rank (Mantel–Cox) testing. For multiple logistic regression, comparison of models was performed with the likelihood ratio test. We reported the hazard ratio (HR) with 95% confidence interval (CI) for each covariate of interest to assess the association between AKI and subsequent mortality. Data analyses were performed with GraphPad Prism (version 8.4.0 for MacOS, GraphPad Software, San Diego, California, USA).

## Results

### Urinary SARS-CoV-2 N Levels at ICU Admission Associate With AKI in COVID-19

**Figure 1:**
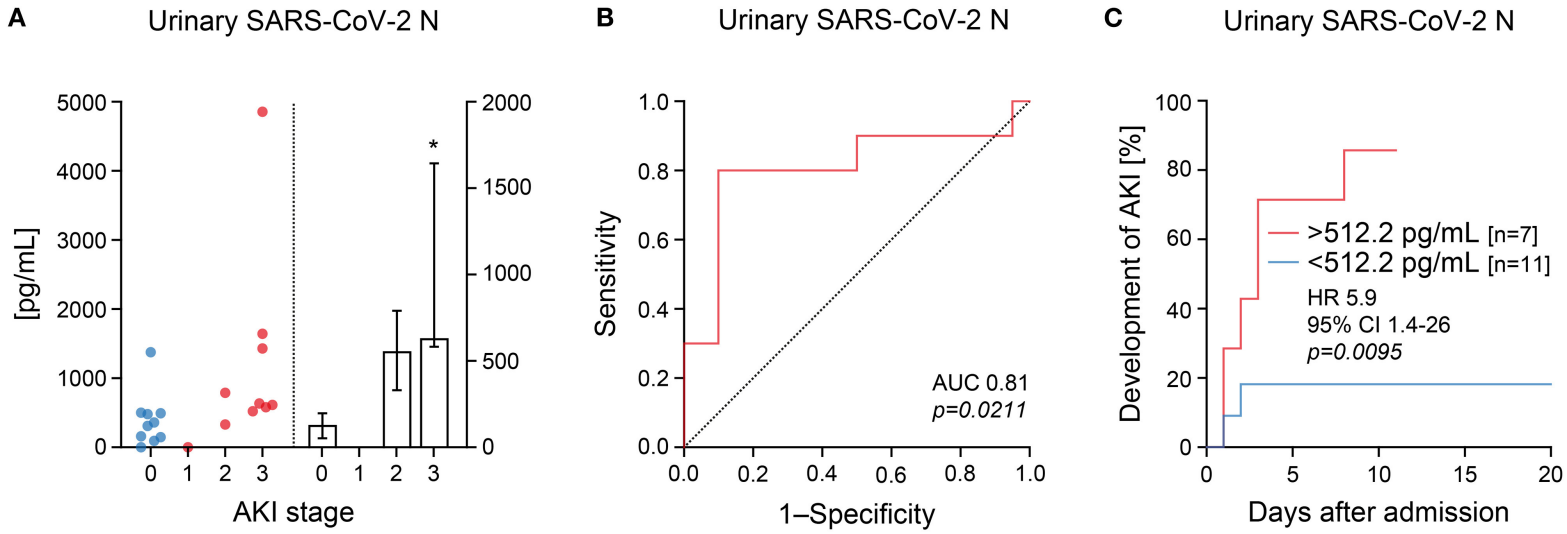
Urinary SARS-CoV-2 N levels at ICU admission associate with AKI in COVID-19. **(A)** Relation between the AKI stage (according to KDIGO) and urinary levels of SARS-CoV-2 N is shown by scatter dot plots (plotted on the left Y-axis) and bar graphs reflecting median with IQR (plotted on the right Y-axis). **(B)** ROC analysis for urinary SARS-CoV-2 N assessed at ICU admission for association with AKI in COVID-19. **(C)** Frequency and survival analysis for cumulative incidence of AKI after group separation for urinary SARS-CoV-2 N at 512.2 pg/mL. AKI, acute kidney injury; AUC, area under the curve; CI, confidence interval; HR, hazard ratio; ICU, intensive care unit; ROC, receiver operator curve; SARS-CoV-2 N, severe acute respiratory syndrome coronavirus 2 nucleocapsid protein.

Among the 20 cases with confirmed SARS-CoV-2 infection requiring ICU supportive care, 10/20 (50%) had AKI of whom 2 presented already at ICU admission and 8 developed AKI within 8 days after admission (Table 1). Interestingly, risk of AKI in severe COVID-19 was not associated with disease severity including respiratory failure at ICU admission (Table 1), implicating specific pathomechanisms that contribute to AKI in COVID-19. The urinary levels of SARS-CoV-2 N, ACE2, and TMPRSS2 were measured at ICU admission (day 1), at day 3, and at day 8 during further clinical course. The AKI stage in COVID-19 was associated with elevated urinary levels of SARS-CoV-2 N measured at ICU admission (Figure 1A, Table 2). A similar association was observed for day 3 and 8 measurements, but the effect was less pronounced (Table 2). The specificity of urinary SARS-CoV-2 N measurements was validated by analyzing an organ failure matched historical ICU cohort with no SARS-CoV-2 infection and AKI in 12/22 (54.5%) of the cases, in which urinary SARS-CoV-2 N was not detectable (Supplementary Table 1) (12). ROC analysis revealed that a cutoff urinary SARS-CoV-2 N level higher than 512.2 pg/mL at ICU admission identified patients with AKI (AUC 0.81, *p* = *0.0211*, Figure 1B). Survival analysis for cumulative incidence of AKI confirmed that urinary SARS-CoV-2 N levels at ICU admission identified patients at risk for AKI (HR 5.9, 95% CI 1.4–26, *p* = *0.0095*, Figure 1C). In contrast, urinary ACE2 or TMPRSS2 levels did not correlate with urinary SARS-CoV-2 N measurements or AKI at any time point (Table 2), implicating that urinary clearance of SARS-CoV-2 N more reflects systemic inflammation due to viral spread rather than renal cell death by direct viral infection of the kidneys. This is further supported by post-mortem analysis from deceased patients with AKI and high urinary SARS-CoV-2 N, where SARS-CoV-2 RNA was not detectable in corresponding kidneys (Supplementary Table 2). These findings suggested that urinary SARS-CoV-2 N occurred independently of shedded cellular membrane proteins essential for viral entry, associated with risk of AKI in severe COVID-19.

**Table 1:** Association between AKI in COVID-19 and clinical findings.

	**AKI**	**No AKI**	***P*-value**
No. of patients (%)	10 (50)	10 (50)	
AKI stage 1—no. (%)	1 (10)	*NA*	
AKI stage 2—no. (%)	2 (20)	*NA*	
AKI stage 3—no. (%)	7 (70)	*NA*	
Onset of AKI (IQR)—days	1.5 (0.75–3)	*NA*	
Age (IQR)—years	69 (64.3–73.3)	71 (58.8–76.5)	0.7241
Female sex—no. (%)	3 (30)	3 (30)	>0.9999
Comorbidities—no. (%)	5 (2–5)	5 (3–6.25)	0.3350
ICU supportive care (IQR)—days	16.5 (12.3–25.8)	11.5 (5.75–18.5)	0.1585
SAPS II (IQR)—points	44 (37.5–60)	38 (35–46.5)	0.3986
SOFA (IQR)—points	9 (7.75–11.3)	8 (4.5–9.5)	0.2295
Heart rate (IQR)—bpm	105 (88.8–116)	78 (58–112)	0.2190
Systolic blood pressure—mmHg	103 (84.8–111)	102 (93–159)	0.3258
Body temperature—°C	38.4 (37.6–38.7)	38.2 (37.2–38.7)	0.7045
P/F—ratio	159 (109–222)	207 (120–261)	0.3150
Death—no. (%)	4 (40)	1 (10)	0.1213

*Median values are shown. AKI, acute kidney injury; bpm, beats per minute; COVID-19: coronavirus disease 2019; ICU, intensive care unit; IQR, interquartile range; No., number; P/F, PaO_2_/FiO_2_ ratio; SAPS II, simplified acute physiology score II; SOFA, sequential organ failure assessment score*.

**Table 2:** Association between AKI in COVID-19 and urinary SARS-CoV-2 N, ACE2, and TMPRSS2.

	**AKI**	**No AKI**	***P*-value**
Urinary ELISA—ICU admission			
No. of urine samples	10	10	
SARS-CoV-2 N (IQR)—pg/mL	624 (475–1,484)	333 (133–464)	**0.0190**
ACE2 (IQR)—pg/mL	0.143 (0.107–2.12)	0.3 (0.195–2.76)	0.2549
TMPRSS2 (IQR)—pg/mL	0.04 (0.023–0.126)	0.111 (0.035–0.35)	0.1713
Urinary ELISA—day 3			
No. of urine samples	10	8	
SARS-CoV-2 N (IQR)—pg/mL	542 (340–902)	381 (40–919)	0.4987
ACE2 (IQR)—pg/mL	0.176 (0.079–1.06)	0.605 (0.138–1.04)	0.2627
TMPRSS2 (IQR)—pg/mL	0.023 (0–0.13)	0.09 (0.021–0.364)	0.1938
Urinary ELISA—day 8			
No. of urine samples	8	5	
SARS-CoV-2 N (IQR)—pg/mL	760 (212–1,024)	110 (0–376)	**0.0435**
ACE2 (IQR)—pg/mL	0.336 (0.164–7.25)	0.2 (0.13–1.59)	0.4584
TMPRSS2 (IQR)—pg/mL	0.055 (0.014–0.445)	0.059 (0.023–0.424)	0.9736

*Median values are shown; bold indicates statistically significant values at the group level. ACE2, angiotensin converting enzyme 2; AKI, acute kidney injury; COVID-19, coronavirus disease 2019; ELISA, enzyme-linked immunosorbent assay; ICU, intensive care unit; IQR: interquartile range; No., number; SARS-CoV-2 N, severe acute respiratory syndrome coronavirus 2 nucleocapsid protein; TMPRSS2, transmembrane protease serine subtype 2*.

### Combining Urinary SARS-CoV-2 N and Plasma Albumin Measurements Identify Patients at Risk for AKI

**Figure 2:**
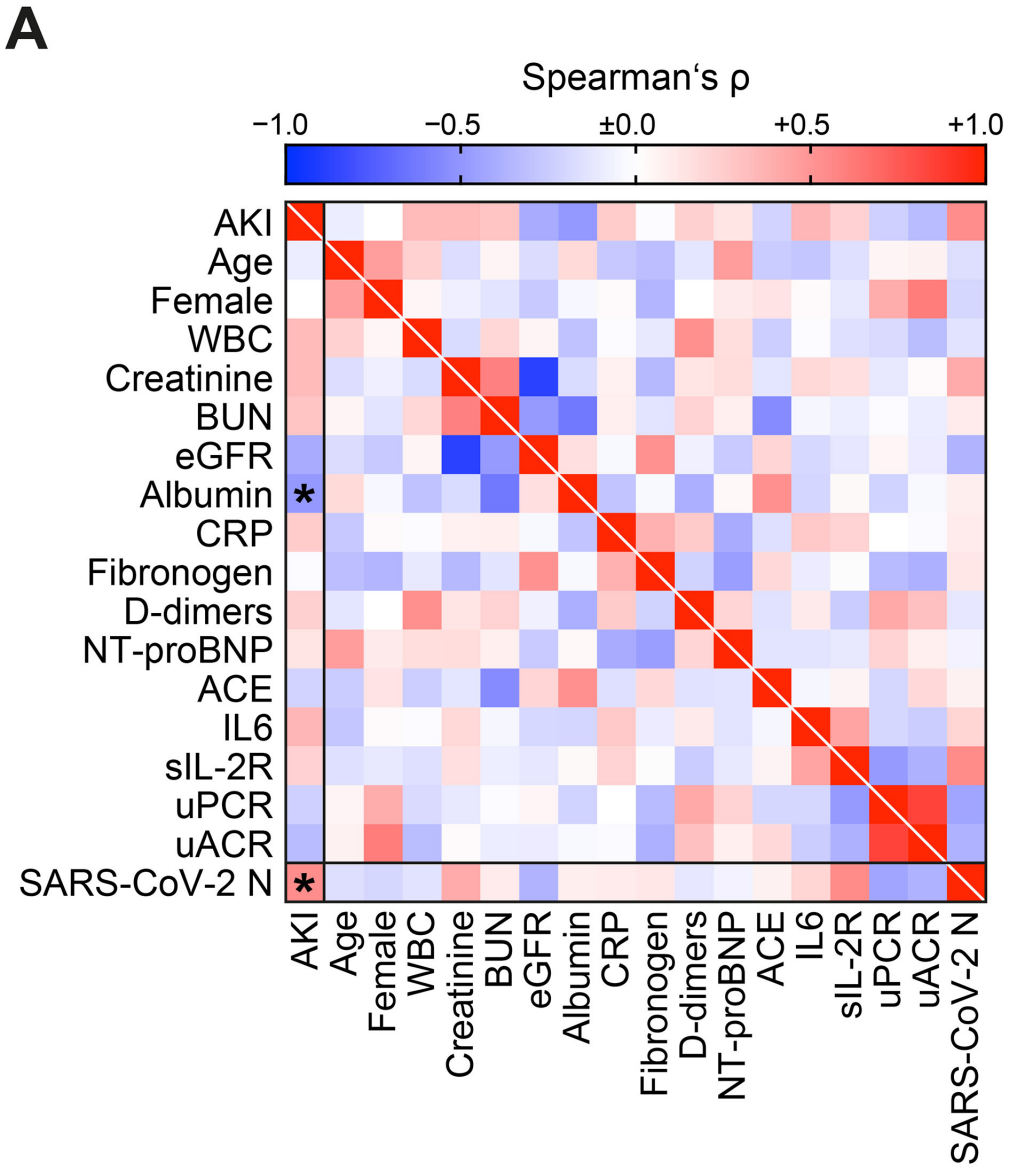
Hypoalbuminemia at time of ICU admission identifies patients at risk for AKI. **(A)** Correlation between AKI, clinical, and routine laboratory parameters assessed at ICU admission is shown by heatmap reflecting the mean values of Spearman's ρ; asterisks indicate *p* < *0.05*. ACE, angiotensin converting enzyme; AKI, acute kidney injury; BUN, blood urea nitrogen; CRP, C-reactive protein; eGFR, estimated glomerular filtration rate (CKD-EPI); IL-6, interleukin-6; NT-proBNP, N-terminal pro-B-type natriuretic peptide; SARS-CoV-2 N, severe acute respiratory syndrome coronavirus 2 nucleocapsid protein; sIL-2R, soluble interleukin-2 receptor; uACR, urinary albumin-to-creatinine ratio; uPCR, urinary protein-to-creatinine ratio; WBC, white blood cells.

**Figure 3:**
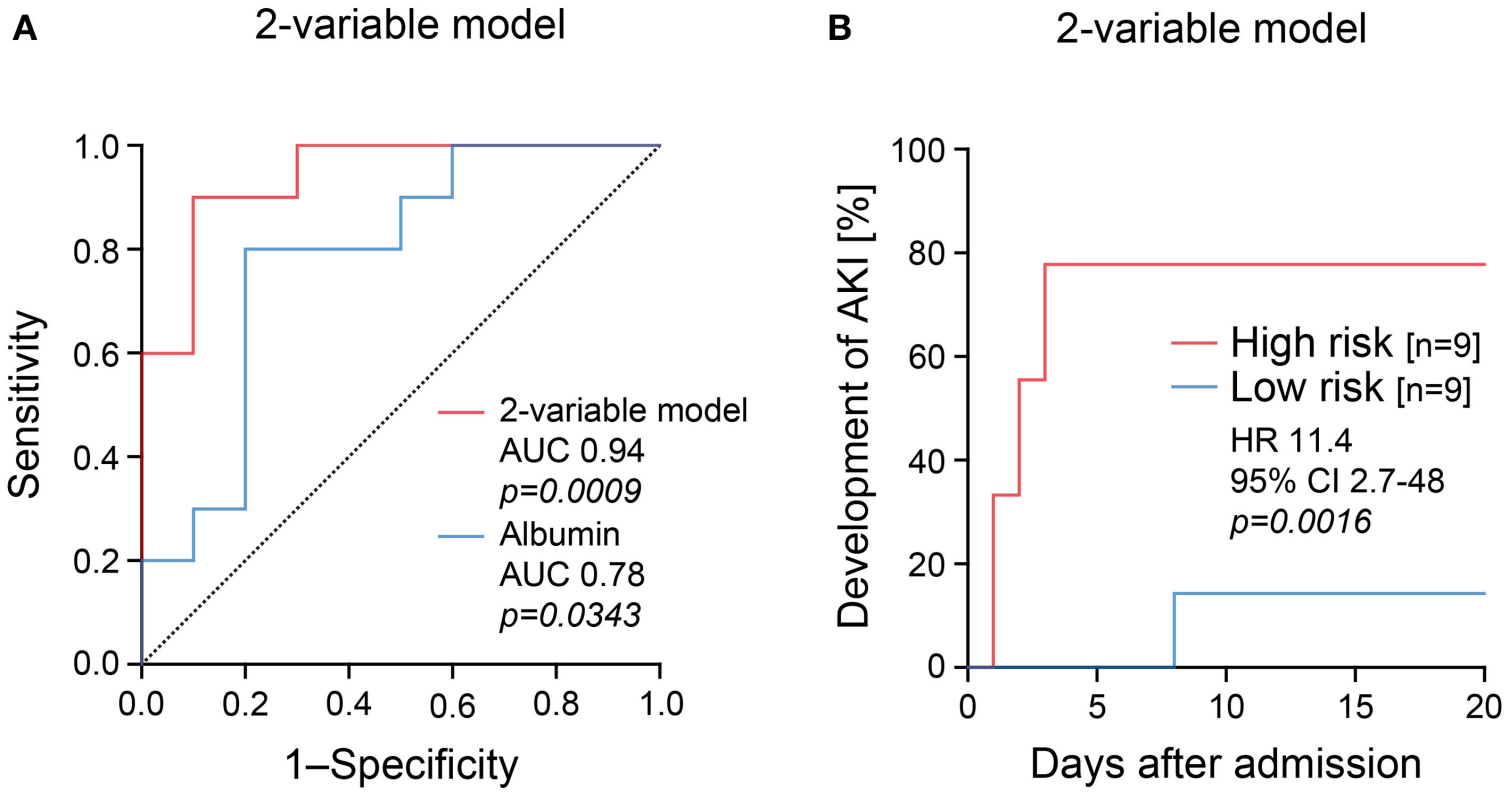
Combining urinary SARS-CoV-2 N and plasma albumin measurements identifies patients at risk for AKI. **(A)** ROC analysis for combining urinary SARS-CoV-2 N and plasma albumin measurements at ICU admission (two-variable model) for association with AKI in COVID-19. **(B)** Frequency and survival analysis for cumulative incidence of AKI after group separation for established two-variable model. AKI, acute kidney injury; AUC, area under the curve, CI: confidence interval; HR, hazard ratio; ROC: receiver operator curve; SARS-CoV-2 N, severe acute respiratory syndrome coronavirus 2 nucleocapsid protein.

Plasma albumin levels at the time of ICU admission have previously been established as a risk marker for AKI and COVID-19 severity (17–19). Therefore, we next compared clinical and routine laboratory parameters assessed at ICU admission for association with AKI. In line with previous findings, hypoalbuminemia at the time of ICU admission also identified patients at risk for AKI in our cohort (Figure 2A, Table 3). Interestingly, the levels of urinary SARS-CoV-2 N and plasma albumin did not correlate directly (Figure 2A), suggesting that combining urinary SARS-CoV-2 N and plasma albumin could further improve risk stratification for AKI in COVID-19. Furthermore, hypoalbuminemia did not correlate with proteinuria including albuminuria (Table 3), indicating that observed hypoalbuminemia reflects systemic inflammation rather than urinary loss. Confirmed by ROC analysis, combining urinary SARS-CoV-2 N higher than 512.2 pg/mL and hypoalbuminemia below 2.05 g/dL (two-variable model, AUC 0.94, *p* = *0.0009*, Figure 3A) outperformed urinary SARS-CoV-2 N alone (AUC 0.81, *p* = *0.0211*, comparison of models: *p* = *0.0016*) or plasma albumin alone (AUC 0.78, *p* = *0.03*, comparison of models: *p* = *0.0061*). Thus, combined urinary SARS-CoV-2 N and plasma albumin levels assessed at ICU admission identified patients at risk for AKI (two-variable model, HR 11.4, 95% CI 2.7-48, *p* = *0.0016*, Figure 3B). In summary, combining urinary SARS-CoV-2 N and plasma albumin levels at ICU admission allowed for a more robust identification of patients at risk for AKI in COVID-19 as compared to analysis of the single markers alone.

**Table 3:** Association between AKI in COVID-19 and laboratory findings at time of ICU admission.

	**AKI**	**No AKI**	***P*-value**
WBC count (IQR)— × 1,000/μL	9.41 (6.76–12.5)	5.64 (4.3–10.9)	0.1655
Serum creatinine (IQR)—μmol/L	90.6 (66.1–151)	76.5 (46.6–92.8)	0.1593
BUN (IQR)—mmol/L	9.1 (5.62–15.4)	6.6 (4.91–9.64)	0.2392
eGFR (IQR)—mL/min/1.73 m^2^	62.3 (39.9–92)	91.4 (73.4–102)	0.0787
Albumin (IQR)—g/dL	1.8 (1.68–2.05)	2.5 (2.08–2.75)	**0.0325**
CRP (IQR)—mg/L	124 (34–220)	87.2 (26.9–133)	0.3150
Fibrinogen (IQR)—mg/dL	379 (306–737)	453 (325–519)	0.9705
D-dimers (IQR)—mg/L	2.12 (0.965–5.9)	1.21 (0.838–2.65)	0.3527
NT-proBNP (IQR)—ng/L	1,258 (252–3,820)	1,061 (183–2,305)	0.6305
ACE (IQR)—IU/L	13.5 (12–22)	18 (12–33.8)	0.3641
IL-6 (IQR)—pg/mL	102 (64.3–418)	37.3 (23.3–90)	0.1431
sIL-2R (IQR)—IU/mL	1,567 (1,283–2,700)	1,386 (573–2,404)	0.3629
uPCR (IQR)—mg/g	354 (240–1,091)	584 (418–1,416)	0.3562
uACR (IQR)—mg/g	83.1 (41.2–174)	242 (61–407)	0.1823

*Median values are shown; bold indicates statistically significant values at the group level. ACE, angiotensin converting enzyme; AKI, acute kidney injury; BUN, blood urea nitrogen; COVID-19, coronavirus disease 2019; CRP, C-reactive protein; eGFR, estimated glomerular filtration rate (CKD-EPI); ICU, intensive care unit; IL-6, interleukin-6; IQR, interquartile range; NT-proBNP, N-terminal pro-B-type natriuretic peptide; sIL-2R, soluble interleukin-2 receptor; uACR, urinary albumin-to-creatinine ratio; uPCR, urinary protein-to-creatinine ratio; WBC, white blood cells*.

### Combining Urinary SARS-CoV-2 N and Plasma Albumin Measurements Associate With Length of ICU Supportive Care and Premature Death in COVID-19

**Figure 4:**
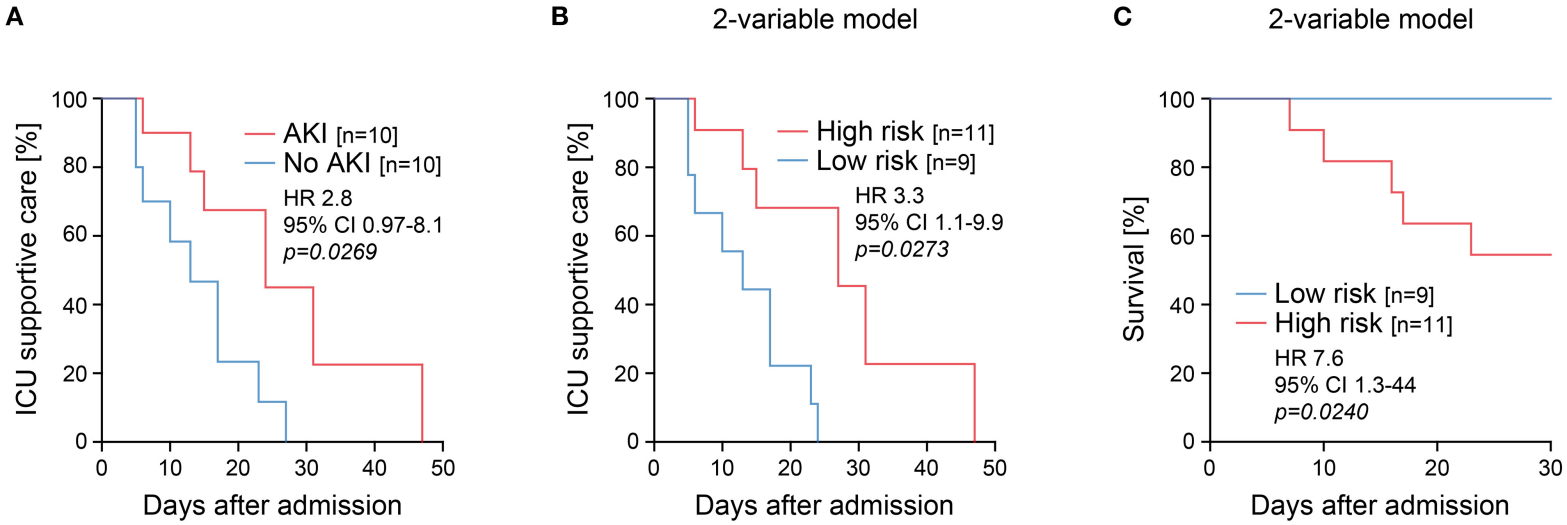
Combining urinary SARS-CoV-2 N and plasma albumin measurements associates with length of ICU supportive care and premature death in COVID-19. **(A)** Duration of ICU supportive care grouped for AKI. **(B)** Duration of ICU supportive care grouped for established two-variable model. **(C)** Premature death in COVID-19 grouped for established two-variable model. AKI, acute kidney injury; CI, confidence interval; HR, hazard ratio; ICU, intensive care unit.

AKI has previously been considered as an independent risk factor for premature death in patients with COVID-19 (1–5). Therefore, we next compared the association between AKI, disease course, outcome, and urinary SARS-CoV-2 N and plasma albumin levels assessed at ICU admission. AKI was associated with a prolonged ICU supportive care before patients could be relocated to a non-ICU medical ward (HR 2.8, 95% CI 0.97–8.1, *p* = *0.0269*, Figure 4A). Urinary SARS-CoV-2 N and plasma albumin measured at ICU admission were equally associated with the length of ICU supportive care (two-variable model, HR 3.3, 95% CI 1.1–9.9, *p* = *0.0273*, Figure 4B). In addition, combining urinary SARS-CoV-2 N and plasma albumin levels at ICU admission identified patients at risk for premature death in COVID-19 (two-variable model, HR 7.6, 95% CI 1.3–44, *p* = *0.0240*, Figure 4C). In summary, AKI was associated with disease severity reflected by prolonged ICU length of stay. Furthermore, urinary SARS-CoV-2 N and plasma albumin levels at ICU admission equally allowed for a robust identification of patients at risk for prolonged ICU length of stay and premature death in COVID-19.

## Discussion

AKI is common in critically ill patients with severe infection and sepsis, associated with mortality rates between 15 and 60% and up to 10 times higher as compared to general ICU cohorts (20–24). Hemodynamic support and guided volume therapy to optimize renal perfusion and to limit intrarenal injury are fundamental preventive strategies. Established laboratory markers to identify AKI do not provide insights into the underlying pathological mechanisms. Based on the assumption that AKI in COVID-19 is caused by direct viral infection of the kidneys, it was unclear whether markers for viral infection (SARS-CoV-2 N) and shedded cellular membrane proteins essential for viral entry (ACE2, TMPRSS2) are detectable in urinary samples of patients with AKI in COVID-19.

Here, we demonstrate for the first time that urinary SARS-CoV-2 N is an early and easily assessable marker to identify patients at risk for AKI and premature death in COVID-19. SARS N has been detected in renal tubules, and SARS-CoV-2 renal tropism has been demonstrated in COVID-19 autopsy studies, suggesting that renal SARS-CoV-2 infection may cause AKI in COVID-19 (7–9). Whether SARS-CoV-2 directly infects the kidneys and related AKI in COVID-19 is induced by direct viral infection of the kidney and responsible for poor outcome or a consequence of systemic viral spread is still controversial (25–28). Our observations that urinary ACE2 and TMPRSS2 do not associate with AKI or disease severity suggest that urinary clearance of SARS-CoV-2 N more reflects systemic inflammation due to viral spread rather than renal cell death by direct viral infection of the kidneys. This is supported by failure to detect SARS-CoV-2 RNA in corresponding kidneys of deceased patients with AKI and high urinary SARS-CoV-2 N and in line with previous studies reporting that urinary SARS-CoV-2 viral load was not more frequently detected in patients who died or developed AKI, suggesting that direct viral infection is unlikely an important mechanism of AKI in COVID-19 (29–31). This is further supported by unspecific post-mortem findings despite multiorgan viral spread in COVID-19 (32).

A variety of potential mechanisms contributing to AKI in COVID-19 have been suggested. The receptor-binding domain of the SARS-CoV-2 spike protein gains entry to host cells by binding to membrane-bound ACE2 that is also present on kidney tubular epithelial cells and podocytes (33, 34). In this context, recent reports suggest that polymorphisms in *ACE2* might alter the ability of SARS-CoV-2 to enter cells in the kidney (33, 34). Furthermore, endothelial dysfunction and microvascular damage indicated by higher levels of D-dimers indicate an important risk factor for coagulopathy associated with COVID-19. Other pro-thrombotic conditions such as thrombotic microangiopathy (TMA) by direct viral activation of the complement system might also contribute to endothelial dysfunction and risk of AKI in patients with COVID-19 (35, 36). In addition, infection with SARS-CoV-2 is associated with induction of an inflammatory response resulting in hyperinflammation resembling a cytokine release syndrome (CRS) that might also contribute to the pathogenesis of multiorgan dysfunction associated with COVID-19, including AKI (37–44). While identification of mechanisms contributing to AKI related to COVID-19 is of relevance and requires further investigation, our findings that urinary SARS-CoV-2 N associates with AKI and COVID-19 severity are also of importance. The accuracy of urinary SARS-CoV-2 N levels to identify patients at risk for AKI in COVID-19 was further improved by plasma albumin measurements, also previously reported as a risk marker for AKI and COVID-19 severity (17–19). Finally, urinary SARS-CoV-2 N and plasma albumin levels identified patients at risk for premature death in COVID-19. Hypoalbuminemia has previously been associated with disease severity and mortality across numerous clinical settings (45). The pathophysiology behind hypoalbuminemia is thought to be secondary to increased capillary permeability, decreased protein synthesis, and decreased half-life of serum albumin, also described in severe COVID-19 (17–19). Furthermore, inflammation may be responsible for the extravasation of serum albumin into the interstitial space due to capillary permeability, with an increased volume distribution of albumin (46). Our observation that hypoalbuminemia did not correlate with proteinuria including albuminuria further supports that hypoalbuminemia in severe COVID-19 is attributed to systemic inflammation and extravasation rather than urinary loss. In summary, our findings that urinary levels of SARS-CoV-2 N associate with AKI and COVID-19 severity might be of great relevance for risk stratification and can potentially lead to early recognition of severe disease to assist clinicians in making informed decision for their patients.

The primary limitation of this study is the small sample size limiting the strength of correlations and the modeling or prediction analyses. Multiple logistic regression was performed as exploratory analyses and should be interpreted with caution due to the study being underpowered. This might limit the results of the multivariate analyses and lead to a certain overfitting. In addition, our findings need further validation in independent cohorts and comparison with other experimental markers of AKI. However, the strong association of urinary SARS-CoV-2 N with AKI and COVID-19 severity in this single-center observational study is promising and requires further investigation.

## Conclusions

In conclusion, urinary SARS-CoV-2 N levels associate with risk for AKI and COVID-19 severity. Therefore, we propose that urinary SARS-CoV-2 N could be used as an early and easily assessable marker to identify patients at risk for AKI and premature death in COVID-19.

## Data Availability Statement

The original contributions presented in the study are included in the article/Supplementary Material, further inquiries can be directed to the corresponding author/s.

## Ethics Statement

The studies involving human participants were reviewed and approved by the institutional review board of the University Medical Center Göttingen, Germany (reference number 25/4/19Ü). The patients/participants provided their written informed consent to participate in this study.

## Author Contributions

BT conceived the study, collected and analyzed data, and wrote the first draft. DT performed urinary ELISA measurements and co-wrote the first draft. HH-W, SP, SK, OM, and MW collected human specimens. MW and BT contributed equally as senior authors. All authors participated in the construction and editing of the manuscript.

## Conflict of Interest

The authors declare that the research was conducted in the absence of any commercial or financial relationships that could be construed as a potential conflict of interest.

## References

[1] Gabarre P Dumas G Dupont T Darmon M Azoulay E Zafrani L. Acute kidney injury in critically ill patients with COVID-19. Intensive Care Med. (2020) 46:1339–48. 10.1007/s00134-020-06153-9 32533197 PMC7290076

[2] Chan L Chaudhary K Saha A Chauhan K Vaid A Zhao S. AKI in hospitalized patients with COVID-19. J Am Soc Nephrol. (2020) 32:151–60. 10.1681/ASN.2020050615 32883700 PMC7894657

[3] Gupta S Coca SG Chan L Melamed ML Brenner SK Hayek SS. AKI treated with renal replacement therapy in critically ill patients with COVID-19. J Am Soc Nephrol. (2020) 32:161–76. 10.1681/ASN.2020060897 33067383 PMC7894677

[4] Xu J Yang X Yang L Zou X Wang Y Wu Y. Clinical course and predictors of 60-day mortality in 239 critically ill patients with COVID-19: a multicenter retrospective study from Wuhan, China. Crit Care. (2020) 24:394. 10.1186/s13054-020-03098-9 32631393 PMC7336107

[5] Joseph A Zafrani L Mabrouki A Azoulay E Darmon M. Acute kidney injury in patients with SARS-CoV-2 infection. Ann Intensive Care. (2020) 10:117. 10.1186/s13613-020-00734-z 32880774 PMC7471244

[6] Nadim MK Forni LG Mehta RL Connor MJ Jr Liu KD. COVID-19-associated acute kidney injury: consensus report of the 25th Acute Disease Quality Initiative (ADQI) Workgroup. Nat Rev Nephrol. (2020) 16:747–64. 10.1038/s41581-020-00356-5 33060844 PMC7561246

[7] Braun F Lutgehetmann M Pfefferle S Wong MN Carsten A Lindenmeyer MT. SARS-CoV-2 renal tropism associates with acute kidney injury. Lancet. (2020) 396:597–8. 10.1016/S0140-6736(20)31759-1 32818439 PMC7431179

[8] Puelles VG Lutgehetmann M Lindenmeyer MT Sperhake JP Wong MN Allweiss L. Multiorgan and renal tropism of SARS-CoV-2. N Engl J Med. (2020) 383:590–2. 10.1056/NEJMc2011400 32402155 PMC7240771

[9] Su H Yang M Wan C Yi LX Tang F Zhu HY. Renal histopathological analysis of 26 postmortem findings of patients with COVID-19 in China. Kidney Int. (2020) 98:219–27. 10.1016/j.kint.2020.04.003 32327202 PMC7194105

[10] Pan XW Xu D Zhang H Zhou W Wang LH Cui XG. Identification of a potential mechanism of acute kidney injury during the COVID-19 outbreak: a study based on single-cell transcriptome analysis. Intensive Care Med. (2020) 46:1114–6. 10.1007/s00134-020-06026-1 32236644 PMC7106051

[11] Hoffmann M Kleine-Weber H Schroeder S Kruger N Herrler T Erichsen S. SARS-CoV-2 cell entry depends on ACE2 and TMPRSS2 and is blocked by a clinically proven protease inhibitor. Cell. (2020) 181:271–80 e8. 10.1016/j.cell.2020.02.052 32142651 PMC7102627

[12] Winkler MS Nierhaus A Holzmann M Mudersbach E Bauer A Robbe L. Decreased serum concentrations of sphingosine-1-phosphate in sepsis. Crit Care. (2015) 19:372. 10.1186/s13054-015-1089-0 26498205 PMC4620595

[13] Le Gall JR Lemeshow S Saulnier F. A new simplified acute physiology score (SAPS II) based on a European/North American multicenter study. JAMA. (1993) 270:2957–63. 10.1001/jama.270.24.2957 8254858

[14] Vincent JL de Mendonca A Cantraine F Moreno R Takala J Suter PM. Use of the SOFA score to assess the incidence of organ dysfunction/failure in intensive care units: results of a multicenter, prospective study. Working group on “sepsis-related problems” of the European Society of Intensive Care Medicine. Crit Care Med. (1998) 26:1793–800. 10.1097/00003246-199811000-00016 9824069

[15] Hoffmann M Mosbauer K Hofmann-Winkler H Kaul A Kleine-Weber H Kruger N. Chloroquine does not inhibit infection of human lung cells with SARS-CoV-2. Nature. (2020) 585:588–90. 10.1038/s41586-020-2575-3 32698190

[16] Corman VM Landt O Kaiser M Molenkamp R Meijer A Chu DK. Detection of 2019 novel coronavirus (2019-nCoV) by real-time RT-PCR. Euro Surveill. (2020) 25:23–30. 10.2807/1560-7917.ES.2020.25.3.2000045 31992387 PMC6988269

[17] Huang J Cheng A Kumar R Fang Y Chen G Zhu Y. Hypoalbuminemia predicts the outcome of COVID-19 independent of age and co-morbidity. J Med Virol. (2020) 92:2152–8. 10.1002/jmv.26003 32406952 PMC7273060

[18] Aziz M Fatima R Lee-Smith W Assaly R. The association of low serum albumin level with severe COVID-19: a systematic review and meta-analysis. Crit Care. (2020) 24:255. 10.1186/s13054-020-02995-3 32456658 PMC7249975

[19] Paliogiannis P Mangoni AA Cangemi M Fois AG Carru C Zinellu A. Serum albumin concentrations are associated with disease severity and outcomes in coronavirus 19 disease (COVID-19): a systematic review and meta-analysis. Clin Exp Med. (2021). 10.1007/s10238-021-00686-z 33511503 PMC7842395

[20] Liano F Pascual J. Epidemiology of acute renal failure: a prospective, multicenter, community-based study. Madrid Acute Renal Failure Study Group. Kidney Int. (1996) 50:811–8. 10.1038/ki.1996.380 8872955

[21] Brivet FG Kleinknecht DJ Loirat P Landais PJ. Acute renal failure in intensive care units–causes, outcome, and prognostic factors of hospital mortality; a prospective, multicenter study. French Study Group on acute renal failure. Crit Care Med. (1996) 24:192–8. 10.1097/00003246-199602000-00003 8605788

[22] Uchino S Kellum JA Bellomo R Doig GS Morimatsu H Morgera S. Acute renal failure in critically ill patients: a multinational, multicenter study. JAMA. (2005) 294:813–8. 10.1001/jama.294.7.813 16106006

[23] Cheng Y Luo R Wang K Zhang M Wang Z Dong L. Kidney disease is associated with in-hospital death of patients with COVID-19. Kidney Int. (2020) 97:829–38. 10.1016/j.kint.2020.03.005 32247631 PMC7110296

[24] Pei G Zhang Z Peng J Liu L Zhang C Yu C. Renal involvement and early prognosis in patients with COVID-19 pneumonia. J Am Soc Nephrol. (2020) 31:1157–65. 10.1681/ASN.2020030276 32345702 PMC7269350

[25] Calomeni E Satoskar A Ayoub I Brodsky S Rovin BH Nadasdy T. Multivesicular bodies mimicking SARS-CoV-2 in patients without COVID-19. Kidney Int. (2020) 98:233–4. 10.1016/j.kint.2020.05.003 32437766 PMC7206432

[26] Khan S Chen L Yang CR Raghuram V Khundmiri SJ Knepper MA. Does SARS-CoV-2 infect the kidney? J Am Soc Nephrol. (2020) 31:2746–8. 10.1681/ASN.2020081229 33051359 PMC7790203

[27] Kudose S Batal I Santoriello D Xu K Barasch J Peleg Y. Kidney biopsy findings in patients with COVID-19. J Am Soc Nephrol. (2020) 31:1959–68. 10.1681/ASN.2020060802 32680910 PMC7461665

[28] Santoriello D Khairallah P Bomback AS Xu K Kudose S Batal I. Postmortem kidney pathology findings in patients with COVID-19. J Am Soc Nephrol. (2020) 31:2158–67. 10.1681/ASN.2020050744 32727719 PMC7461662

[29] Frithiof R Bergqvist A Jarhult JD Lipcsey M Hultstrom M. Presence of SARS-CoV-2 in urine is rare and not associated with acute kidney injury in critically ill COVID-19 patients. Crit Care. (2020) 24:587. 10.1186/s13054-020-03302-w 32993742 PMC7523248

[30] Akilesh S Nast CC Yamashita M Henriksen K Charu V Troxell ML. Multicenter clinicopathologic correlation of kidney biopsies performed in COVID-19 patients presenting with acute kidney injury or proteinuria. Am J Kidney Dis. (2020) 77:82–93.e1. 10.1053/j.ajkd.2020.10.001 33045255 PMC7546949

[31] Nasr SH Alexander MP Cornell LD Herrera LH Fidler ME Said SM. Kidney biopsy findings in patients with COVID-19, kidney injury, and proteinuria. Am J Kidney Dis. (2020) 77:465–8. 10.1053/j.ajkd.2020.11.002 33217501 PMC7671921

[32] Remmelink M De Mendonca R D'Haene N De Clercq S Verocq C Lebrun L. Unspecific post-mortem findings despite multiorgan viral spread in COVID-19 patients. Crit Care. (2020) 24:495. 10.1186/s13054-020-03218-5 32787909 PMC7422463

[33] Delanghe JR Speeckaert MM De Buyzere ML. The host's angiotensin-converting enzyme polymorphism may explain epidemiological findings in COVID-19 infections. Clin Chim Acta. (2020) 505:192–3. 10.1016/j.cca.2020.03.031 32220422 PMC7102561

[34] Othman H Bouslama Z Brandenburg JT da Rocha J Hamdi Y Ghedira K. Interaction of the spike protein RBD from SARS-CoV-2 with ACE2: similarity with SARS-CoV, hot-spot analysis and effect of the receptor polymorphism. Biochem Biophys Res Commun. (2020) 527:702–8. 10.1016/j.bbrc.2020.05.028 32410735 PMC7221370

[35] Goldstein SL Dahale D Kirkendall ES Mottes T Kaplan H Muething S. A prospective multi-center quality improvement initiative (NINJA) indicates a reduction in nephrotoxic acute kidney injury in hospitalized children. Kidney Int. (2020) 97:580–8. 10.1016/j.kint.2019.10.015 31980139

[36] Noris M Benigni A Remuzzi G. The case of complement activation in COVID-19 multiorgan impact. Kidney Int. (2020) 98:314–22. 10.1016/j.kint.2020.05.013 32461141 PMC7246017

[37] Mehta P McAuley DF Brown M Sanchez E Tattersall RS Manson JJ. COVID-19: consider cytokine storm syndromes and immunosuppression. Lancet. (2020) 395:1033–4. 10.1016/S0140-6736(20)30628-0 32192578 PMC7270045

[38] Ritchie AI Singanayagam A. Immunosuppression for hyperinflammation in COVID-19: a double-edged sword? Lancet. (2020) 395:1111. 10.1016/S0140-6736(20)30691-7 32220278 PMC7138169

[39] Moore JB June CH. Cytokine release syndrome in severe COVID-19. Science. (2020) 368:473–4. 10.1126/science.abb8925 32303591

[40] Yang X Yu Y Xu J Shu H Xia J Liu H. Clinical course and outcomes of critically ill patients with SARS-CoV-2 pneumonia in Wuhan, China: a single-centered, retrospective, observational study. Lancet Respir Med. (2020) 8:475–81. 10.1016/S2213-2600(20)30079-5 32105632 PMC7102538

[41] Xu Z Shi L Wang Y Zhang J Huang L Zhang C. Pathological findings of COVID-19 associated with acute respiratory distress syndrome. Lancet Respir Med. (2020) 8:420–2. 10.1016/S2213-2600(20)30076-X 32085846 PMC7164771

[42] Winkler MS Korsten P Binder C Tampe B. Correspondence on 'Interleukin-6 receptor blockade with subcutaneous tocilizumab in severe COVID-19 pneumonia and hyperinflammation: case-control study'. Ann Rheum Dis. (2020). 10.1136/annrheumdis-2020-218836 32958511

[43] Caricchio R Gallucci M Dass C Zhang X Gallucci S Fleece D. Preliminary predictive criteria for COVID-19 cytokine storm. Ann Rheum Dis. (2021) 80:88–95. 10.1136/annrheumdis-2020-218323 32978237

[44] Tampe D Winkler MS Korsten P Hakroush S Moerer O Tampe B. Correspondence on 'Preliminary predictive criteria for COVID-19 cytokine storm'. Ann Rheum Dis. (2021). 10.1136/annrheumdis-2020-219709 33414185

[45] Akirov A Masri-Iraqi H Atamna A Shimon I. Low albumin levels are associated with mortality risk in hospitalized patients. Am J Med. (2017) 130:1465 e11– e19. 10.1016/j.amjmed.2017.07.020 28803138

[46] Soeters PB Wolfe RR Shenkin A. Hypoalbuminemia: pathogenesis and clinical significance. JPEN J Parenter Enteral Nutr. (2019) 43:181–93. 10.1002/jpen.1451 30288759 PMC7379941

