# Supplementary material for: Urinary Levels of SARS-CoV-2 Nucleocapsid Protein Associate With Risk of AKI and COVID-19 Severity: A Single-Center Observational Study
Source: Front Med (Lausanne). 2021 May 25;8:644715. doi: 10.3389/fmed.2021.644715 (PMC8185060; doi:10.3389/fmed.2021.644715)
Supplement: Supplementary file 1 [file Data_Sheet_1.PDF]

# Urinary levels of SARS-CoV-2 nucleocapsid protein associate with risk of AKI and COVID-19 severity: A single-center observational study

## Supplementary Material

Désirée Tampe[1], Samy Hakroush[2], Mark-Sebastian Bösherz[2], Jonas Franz[3-5], Heike Hofmann-Winkler[6], Stefan Pöhlmann[6,7], Stefan Kluge[8], Onnen Moerer[9], Christine Stadelmann[3], Philipp Ströbel[2], Martin Sebastian Winkler[9*], Björn Tampe[1*]

[1]*Department of Nephrology and Rheumatology, University Medical Center Göttingen, Germany*

[2]*Institute of Pathology, University Medical Center Göttingen, Germany*

[3]*Institute of Neuropathology, University Medical Center Göttingen, Germany*

[4]*Max Planck Institute for Experimental Medicine, Germany*

[5]*Campus Institute for Dynamics of Biological Networks, University of Göttingen, Germany*

[6]*Infection Biology Unit, German Primate Center, Leibniz Institute for Primate Research Göttingen, Germany*

[7]*Faculty of Biology and Psychology, University Göttingen, Germany*

[8]*Department of Intensive Care Medicine, University Medical Center Hamburg-Eppendorf, Germany*

[9]*Department of Anesthesiology, Emergency and Intensive Care Medicine, University Medical Center Göttingen, Germany*

[*]*Contributed equally as senior authors*

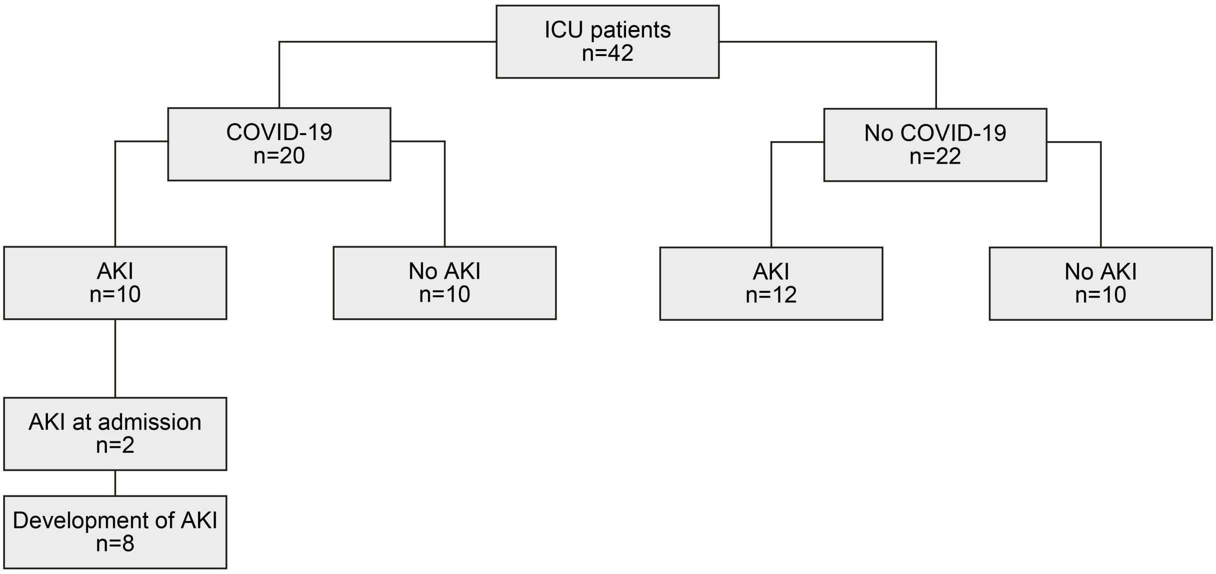


**Supplementary Figure 1.** Strengthening the Reporting of Observational Studies in Epidemiology (STROBE) flow chart of patient disposition.

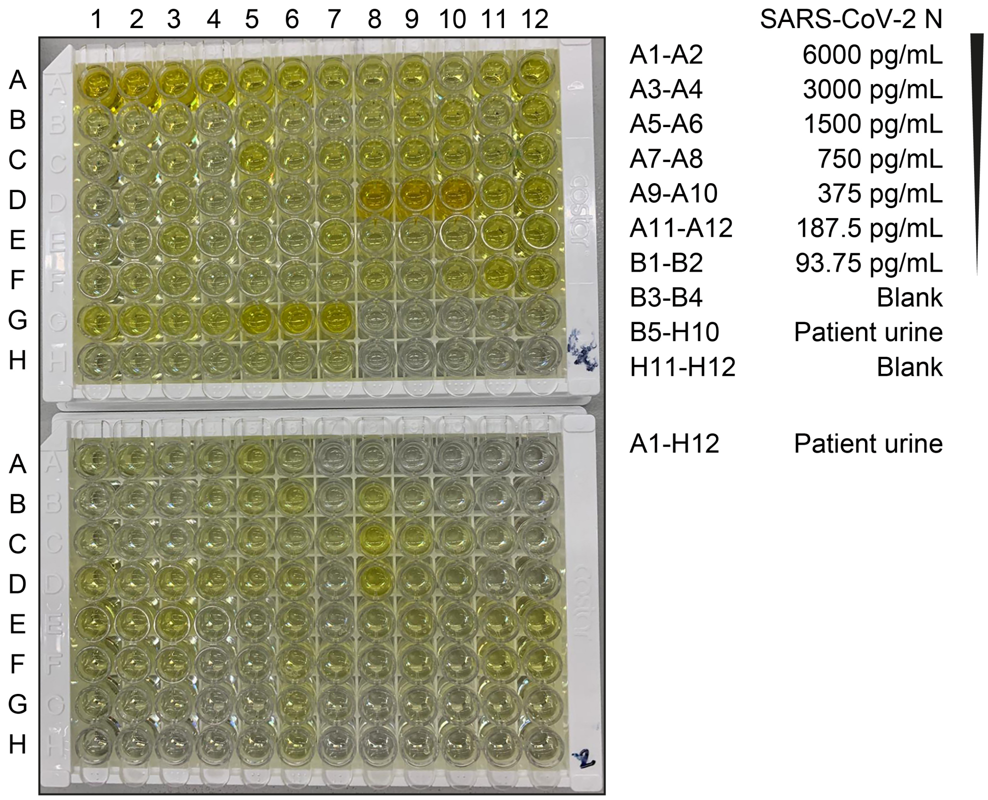


**Supplementary Figure 2.** Photomicrographs of urinary SARS-CoV-2 N ELISA measurements in 20 cases with confirmed SARS-CoV-2 infection. ELISA, enzyme-linked immunosorbent assay; SARS-CoV-2 N, severe acute respiratory syndrome coronavirus 2 nucleocapsid protein.

**Supplementary Table 1. Clinical and laboratory findings at time of ICU admission.**

| | *COVID-19* | *No COVID-19* | *P value* |
|---|---|---|---|
| *General data* | | | |
| No. of patients (%) | 20 | 22 | |
| AKI– no. (%) | 10 (50) | 12 (54.5) | *0.7683* |
| Age (IQR) – years | 71 (62.3-74.8) | 65 (53.3-74) | *0.0785* |
| Female sex – no. (%) | 6 (30) | 9 (40.9) | *0.4612* |
| *Laboratory findings* | | | |
| Serum creatinine (IQR) – μmol/L | 79.6 (60.1-107.6) | 154.3 (76.9-232.7) | *0.0197* |
| BUN (IQR) – mmol/L | 19.5 (15.3-29.8) | 27.5 (16.5-40.3) | *0.3516* |
| *Urinary ELISA – ICU admission* | | | |
| SARS-CoV-2 N (IQR) – pg/mL | 496.6 (198.4-751.7) | ND | |

Median values are shown. AKI, acute kidney injury. BUN, blood urea nitrogen. ICU, intensive care unit. IQR, interquartile range. ND, not detectable. No., number. SARS-CoV-2 N, severe acute respiratory syndrome coronavirus 2 nucleocapsid protein.

**Supplementary Table 2. Detection of SARS-CoV-2 RNA in post-mortem kidneys.**

| | | | | | *SARS-CoV-2 RNA* | | | |
|---|---|---|---|---|---|---|---|---|
| *ID* | *Age* | *Female* | *AKI* | *Urinary SARS-CoV-2 N pg/mL* | *Left cortex* | *Right cortex* | *Left medulla* | *Right medulla* |
| 8 | 80 | 0 | 1 | 4855.2 | ND | ND | ND | ND |
| 17 | 67 | 0 | 1 | 523.4 | ND | ND | ND | ND |

AKI: acute kidney injury. ND: not detectable. SARS-CoV-2 N: severe acute respiratory syndrome coronavirus 2 nucleocapsid protein.
